# *Beauveria bassiana* delivered through a cellulose-based hydrogel is effective against the red poultry mite, *Dermanyssus gallinae*

**DOI:** 10.1007/s10493-024-00993-6

**Published:** 2025-02-27

**Authors:** Ifra Siddique, Antonio Camarda, Marco Friuli, Wafa Rhimi, Elena Circella, Nicola Pugliese, Christian Demitri, Giovanni Benelli, Domenico Otranto, Claudia Cafarchia

**Affiliations:** 1 Department of Veterinary Medicine, University of Bari Aldo Moro, S.P. per Casamassima, km 3, Valenzano, BA 70010 Italy; 2 Department of Engineering for Innovation, University of Salento, Lecce, Italy; 3 Department of Experimental Medicine, University of Salento, Lecce, Italy; 4 Department of Agriculture, Food and Environment, University of Pisa, Pisa, Italy; 5 Department of Veterinary Clinical Sciences, City University of Hong Kong, Hong Kong, China

**Keywords:** Biological control, Dermanyssidae, Entomopathogenic fungi, Hydroxyethyl cellulose-based hydrogel, Conidial suspension

## Abstract

Though the entomopathogenic fungus *Beauveria bassiana* has shown to be efficacious for managing *Dermanyssus gallinae* infestations, its delivery as liquid formulation poses concerns related to environmental stability and efficacy. To overcome such concerns, here we proposed a hydrogel matrix based on hydroxyethyl-cellulose (HEC) for delivering *B. bassiana* to control *D. gallinae*. Nymph and adult mites were exposed to a hydrogel matrix or filter papers containing *B. bassiana* (10^7^ conidia/mL), with and without a pre-incubation period of 4 days at 25 °C (treated groups). As control groups, mites were exposed to hydrogel matrix or filter papers with sterile distilled water. Results showed higher mortality of all stages of *D. gallinae* in treated groups compared to respective control groups. The LT_50_ and LT_90_ estimated on *D. gallinae* were lower in treated groups (LT_50_ ≤ 9.5 days for adults; and LT_50_ ≤ 10.7 days for nymphs; LT_90_ ≤ 14.9 for adults; LT_90_ ≤ 17.9 days for nymphs) than in control groups (LT_5 0_ ≥14.9 days for adults, LT_50_ > 19 days for nymphs; LT_90_ > 20 days for adults and nymphs). Pre-incubation significantly reduced LT_50_ and LT_90_ with respect to other treated groups. Overall, our study outlined that the conidial suspension of *B. bassiana* in hydrogel is efficacious against both nymphs and adults of *D. gallinae*. The pre-incubation of *B. bassiana* in the HEC enhanced its efficacy. Thus, delivering *B. bassiana* through HEC-based hydrogel matrix may represent an effective and sustainable solution for managing *D. gallinae* infestations in the poultry industry.

## Introduction

Due to its blood-sucking habits, *Dermanyssus gallinae* De Geer (1778) (Mesostigmata: Dermanyssidae), also known as the poultry red mite (PRM), causes deleterious impact on both productivity and welfare of the animals (Schiavone et al. 2022), with significant economic losses (De Luna et al. 2008; Kasburg et al. 2016; Schiavone et al. 2022). In addition, PRM may act as a fomite and vector for several pathogenic zoonotic agents, including *Escherichia coli * Migula, *Salmonella* spp., *Enterococcus gallinarum* Bridge and Sneat, and influenza virus (Schiavone et al. 2022). The impact of PRM infestation in poultry farms is expected to increase in the future due to several factors, such as the transformation of housing systems in laying hen husbandry, climate change, and lack of effective control measures; A limited number of acaricidal drugs are currently available for managing this infestation in poultry farms (Sigognault Flochlay et al. 2017), chiefly fluralaner, but also phoxim, an organophosphate, while other substances, such as carbamates and pyrethroids are not authorized in the UE (Marangi et al. 2012; Abbas et al. 2013; Sigognault Flochlay et al. 2017). Nonetheless, the repeated long-term use of those drugs and their inappropriate dosing have led to reduced acaricide efficacy against red mite populations (Abbas et al. 2013; Schiavone et al. 2023), raising major concerns in the authorities for human consumers. Considering the withdrawal or ban from European markets of several products (Sigognault Flochlay et al. 2017), the interest of the scientific community, the layer industry, and the European Union was strongly oriented towards innovative treatment approaches to control the PRM.

In this scenario, the use of entomopathogenic fungi (EPF), such as *Aspergillus oryzae* (Ahlb.) Cohn 1884, *Beauveria bassiana* (Bals.-Criv.) Vuill. 1912 and *Metarhizium anisopliae* (Metschn.) Sorokīn 1883, is recognized as an alternative biocontrol tool that may overcome the employ of chemical acaricides and insecticides (Immediato et al. 2015; Tomer et al. 2018; Wang et al. 2019; Shang et al. 2022; Ruiu et al. 2024). Noteworthy, *B. bassiana* has shown high effectiveness in controlling *D. gallinae* (Immediato et al. 2015, 2016; de Oliveira et al. 2020; Park et al. 2022; Truong et al. 2024) and *Rhipicephalus sanguineus* sensu lato (Cafarchia et al. 2015), though its limited viability in the environment is hampering its large usage under field conditions (Cafarchia et al. 2022). To develop delivery systems for EPF that guarantee the survival of spores in a specific matrix without compromising their virulence, hydrogels based on natural polymers technology have been developed and tested in different contexts for delivery drugs or EPF (Cafarchia et al. 2022; Hartzke et al. 2022). Due to their excellent material, biocompatibility, and biodegradability, the association of biopolymers with biocides has been proposed as an optimized lure to obtain cost-competitive, safe, and eco-friendly pest control tools (Chen 2020; Cafarchia et al. 2022; Hartzke et al. 2022). Recently, hydroxyethyl cellulose (HEC)-based hydrogel has proven its suitability as a growth substrate of *B. bassiana* (Friuli et al. 2021). In the present study, we investigated the efficacy of HEC as a delivery system for *B. bassiana* for the control of nymphs and adults of *D. gallinae*, also assessing the potential impact of a pre-incubation period of 4 days at 25 °C.

## Materials and methods

### Mite collection

*Dermanyssus gallinae* specimens were collected from two laying hen farms (Altamura; 40.844897, 16.535471; Bitritto, 41.040263, 16.837238) in the province of Bari (Apulia, southern Italy). The farms were naturally infested by the PRM and no acaricide treatments were administered during the two months before the collection. After collection, mites were carefully stored in sealed plastic bags and transported to the Department of Veterinary Medicine, University of Bari, to be morphologically identified following the keys and atlas by Moss (1968) and Di Palma et al. (2012), respectively. Adults and nymphs (i.e., protonymphs and deutonymphs) were then separated and starved in the dark at 20 °C for 5 days prior to being used in the experiments.

### *Beauveria bassiana* strain

A locally isolated strain of *B. bassiana* (strain CD1123) was obtained from naturally infected *R. sanguineus* sensu lato adult ticks collected in a private dog shelter in Putignano (Bari, southern Italy), and morphologically and molecularly identified as previously described (Cafarchia et al. 2015). The *B. bassiana* strain was maintained on potato dextrose agar (PDA; Liofilchem, Roseto degli Abruzzi, Italy) and kept at 4 °C. The conidial infection suspension (CIS) of *B. bassiana* was obtained by culturing *B. bassiana* on PDA for three weeks at 26 °C. To obtain enough material, 15 replicate cultures were set up contemporarily. Conidia were harvested by washing the plates with sterile distilled water containing 0.1% peptone and 0.1% tween 80 (Cafarchia et al. 2015). Turbidity was adjusted spectrophotometrically to a McFarland optical density of 6.5, corresponding to 1–5 × 10^7^ conidia/mL (Immediato et al. 2015) using a densitometer DEN 1 (Biosan, Riga, Latvia). The conidial quantity was then assessed through quantitative plate counts of colonies on PDA.

### Dry hydrogel preparation

Hydrogel was prepared at the facilities of the University of Salento (Lecce, Italy) by adding 12 g of sterile hydroxyethyl-cellulose powder (HEC), average molecular weight 720,000; Merck, Milan, Italy) and 3 g of sorbitol as humectant in 100 mL of distilled water. The mixture was stirred at room temperature for 30 min to obtain a clear gel. The gel was then poured on a frozen distilled water surface to obtain a 3–4 mm layer, which was frozen and freeze-dried under a vacuum to remove water. The dried gel layer was cut into three pieces and then UV sterilized for 30 min (Fiuli et al. 2021).

### Laboratory bioassays

Two bioassays were performed to assess the efficacy of *B. bassiana* delivered through a cellulose-based hydrogel. In the first bioassay, a total of 960 mites (i.e., 480 adults and 480 nymphs), were separated in four different groups, two control (CG1 and CG2) and two treated (TG1 and TG2) groups. Each group consisted of four subgroups of 30 mites to better assess the mite mortality. The mites were placed into bioassay rooms (BR), consisting of filter paper (Whatman no. 1, 10 × 10 mm Labor, 67 g/m^2^, Tecnochimica Moderna, Italy) or HEC disk on Petri dishes (diameter 60 mm) with (TG) and without *B. bassiana* CIS (CG) (see Table 1). Briefly, for CG1 and TG1, Petri dishes contained HEC disk of the same diameter of the plate, while for CG2 and TG2, Petri dishes contained filter papers (FPs). HEC and FP were coated with 0.2 mL of *B. bassiana* CIS (10^7^ conidia/mL) in TG1 and TG2, or with 0.2 mL of sterile peptone water plus 0.1% Tween 80 in CG1 and CG2, groups, respectively. The mites were then positioned on BRs and the Petri dishes were incubated (25 ± 1 °C and RH = 80 ± 5%) after being covered with a lid and sealed with parafilm.

The second bioassay was prepared in the same way by “pre-incubating” the bioassay rooms at 25 °C for 4 days after coating HEC-based hydrogel (ITG1) and FP (ITG2) with 0.2 mL of *B. bassiana* CIS (10^7^ conidia/mL) or with 0.2 mL of sterile distilled water containing 0.1% peptone and 0.1% tween 80 (ICG1 and ICG2, respectively). After the pre-incubation period, a total of 480 adults and 480 nymphs were tested and mites were added in the bioassay rooms. As for the first bioassay, each group consisted of four subgroups of 30 mites. Also, in this case, the bioassay rooms were incubated at a temperature of 25 ± 1 °C and 80 ± 5% RH after being covered with a lid and sealed with parafilm.

The scheme of the experiments is summarized in Table 1.

**Table 1:** Bioassay room composition for testing the effectiveness of *Beauveria bassiana* (*bb*) delivered as conidia suspension on filter paper (FP) and on hydroxyethyl cellulose-based hydrogel (HEC) on adults and nymphs of *Dermanyssus gallinae*

Dermanyssus gallinae groups	Bioassay room composition
Treated Group 1 (TG1)	HEC + *Bb* in sterile peptone water plus 0.1% Tween 80.
Treated Group 2 (TG2)	FP + *Bb* in sterile peptone water plus 0.1% Tween 80
Control Group 1 (CG1)	HEC + sterile peptone water plus 0.1% Tween 80
Control Group 2 (CG2)	FP + sterile peptone water plus 0.1% Tween 80
Incubated Treated Group 1 (ITG1)	Pre-incubated HEC + *Bb* in sterile peptone water plus 0.1% Tween 80
Incubated Treated Group 2 (ITG2)	Pre-incubated FP + *Bb* in sterile peptone water plus 0.1% Tween 80
Incubated Control Group 1 (ICG1)	Pre-incubated HEC + sterile peptone water plus 0.1% Tween 80
Incubated Control Group 2 (ICG2)	Pre-incubated FP + sterile peptone water plus 0.1% Tween 80

In both bioassays, PRM mortality was evaluated daily. Mites were considered dead if they exhibited no movement after repeated mechanical stimulation with an entomological pin by three different examiners. The death caused by fungal infection was checked according to Koch’s postulate and a dead mite for each group was cultured on PDA to verify the presence of viable fungus. All experiments were repeated in triplicate, and dead mites were not removed from the bioassay room.

### Statistical analysis

For each group, times needed to kill 50% and 90% of the exposed population (LT_50_ and LT_90_, respectively) were calculated using probit analysis with the confidence level set at 0.95 and expressed in days as LT ± standard error (SE). When probit analysis returned LT_50_ or LT_90_ higher than 20 days, they were indicated as > 20.

The pairwise comparison of lethal times was carried out by applying the ratio test described in Wheeler et al. (2006). The LT ratios were considered significantly different by 1 when *p* < 0.05. All the analyses were performed using R v. 4.4.2.

## Results

**Fig. 1:**
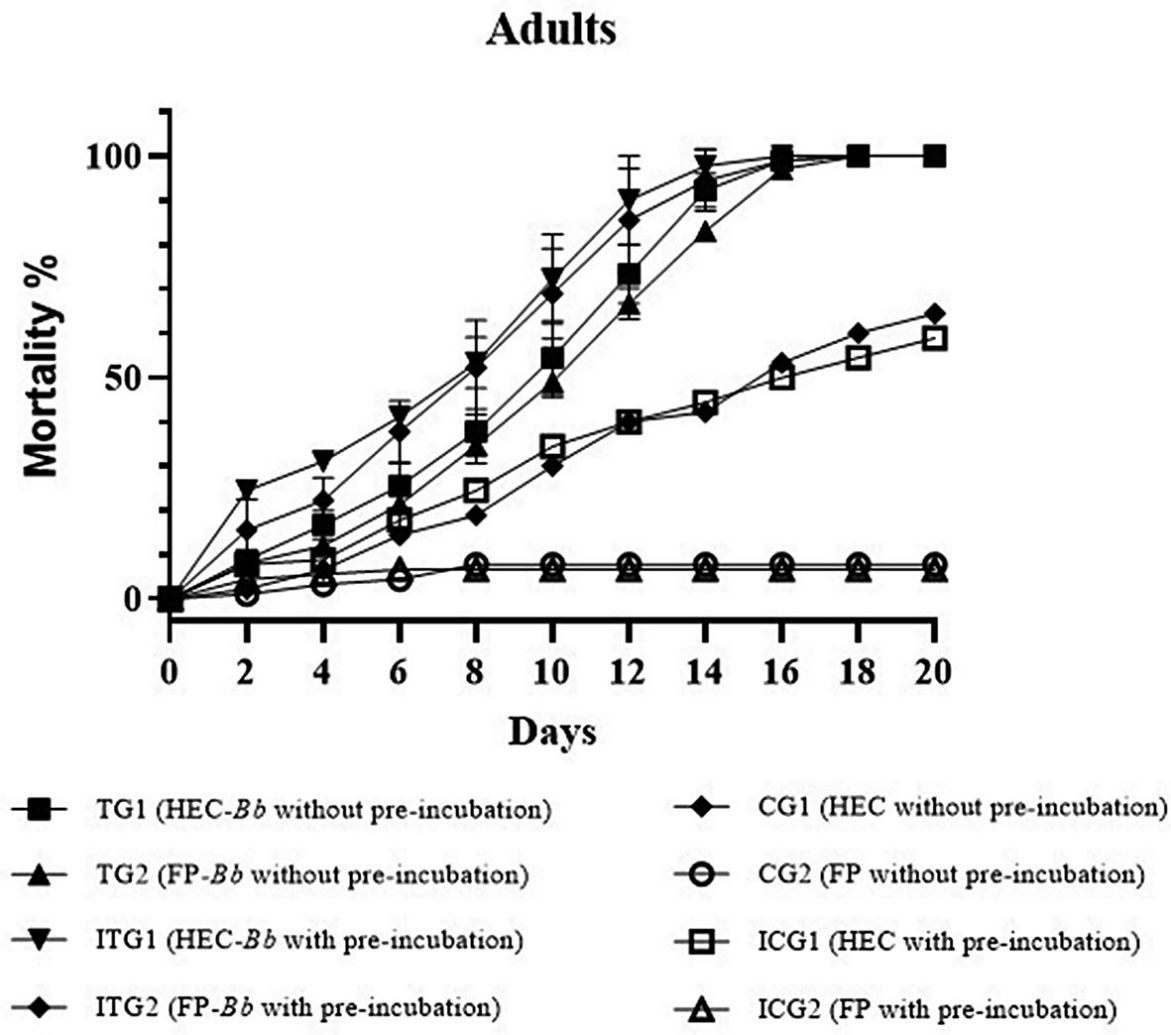
Mortality rates of *Dermanyssus gallinae* adults in treated and control groups with and without pre-incubation

**Fig. 2:**
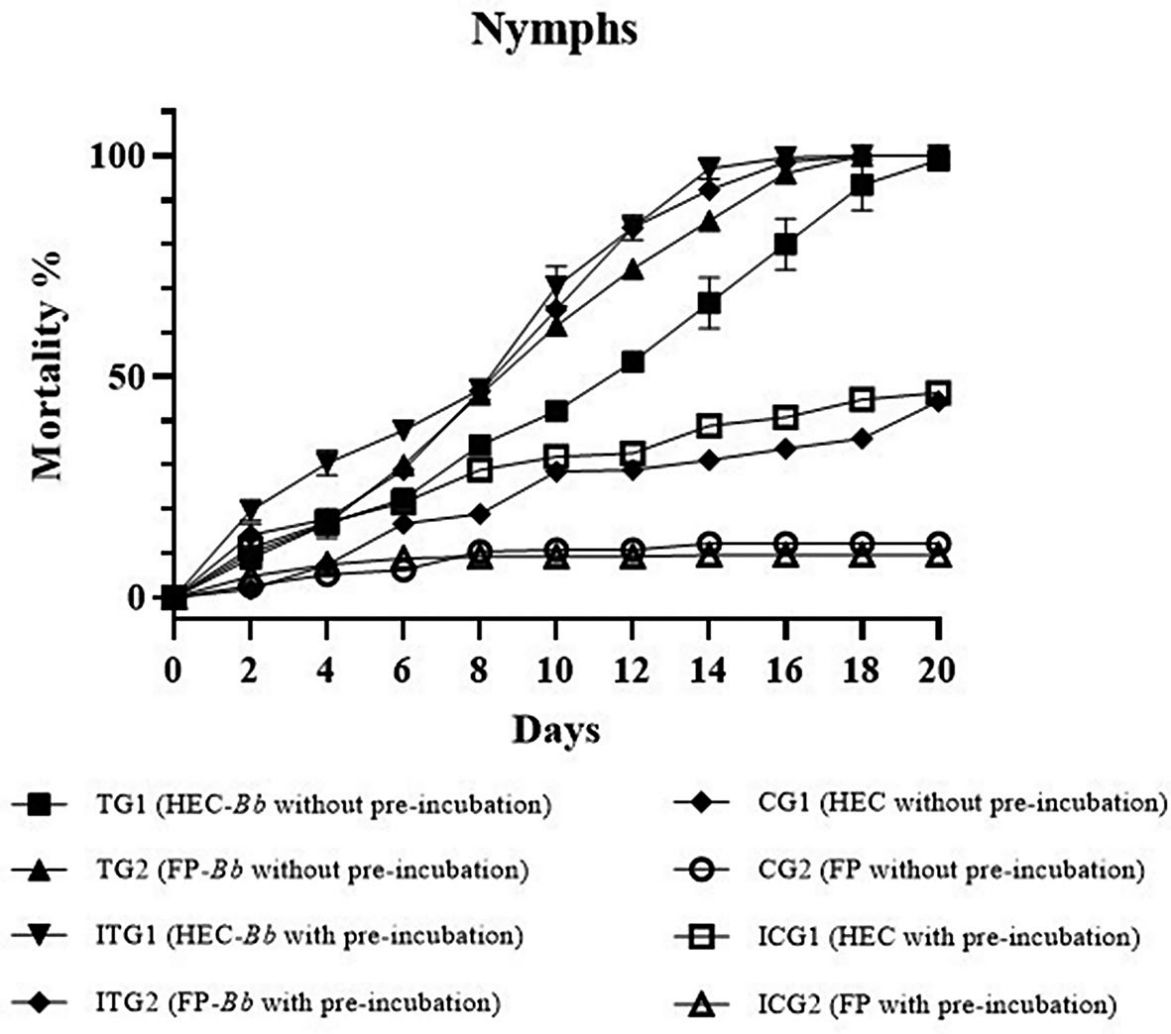
Mortality rates of *Dermanyssus gallinae* nymphs in treated and control groups with and without pre-incubation

The mortality rates of adults and nymphs of *D. gallinae* when exposed to *B. bassiana* applied through HEC or on filter paper, with and without pre-incubation, are reported in Figs. 1 and 2. Mortality of *D. gallinae* was significantly higher, for all the tested stages, in the treated groups compared to the respective control groups (adults: *p* < 0.0001; nymphs: *p* < 0.0001). Among treated groups, mite mortality was higher at all stages in pre-incubated groups ITG1 and ITG2 than in those without pre-incubation (i.e., TG1 and TG2). A higher mortality rate was recorded for all stages in CG1 and ICG1, in which HEC disks were settled, if compared to those recorded in CG2 and ICG2, which contained FP.

The LT_50_ for adults was 8.8. ± 0.1 days and 9.5 ± 0.1 in TG1 (*B. bassiana* administered through HEC-based hydrogel) and TG2 (*B. bassiana* administered through FP), respectively, while it was 6.7 ± 0.1 days and 7.4 ± 0.1 in ITG1 and ITG2, respectively, where *B. bassiana* was applied after pre-incubation (Table 2). LT_90_ was 14.0 ± 0.2 days and 14.9 ± 0.2 days in TG1 and TG2, respectively, and higher than 20 days in CG1 and in CG2, while it was 12.2 ± 0.2 days and 12.7 ± 0 days in ITG1 and ITG2, respectively (Table 2). In all cases, values related to TGs were significantly lower than in CGs (Table 2). The same trend was observed for nymphs, for which LT_50_ was 10.7 ± 0.2 days and 8.6 ± 0.1 in TG1 and TG2, respectively, 7.2 ± 0.1 days and 8.0 ± 0.1 days in ITG1 and ITG2, while it was higher than 20 days in CG1, CG2, and ICG2, and 19 ± 0.5 days ICG1 (Table 2). For nymphs, LT_90_ was 17.9 ± 0.3 days and 14.4 ± 0.2 days in TG1 and TG2, respectively, 12.7 ± 0.2 days and 13.3 ± 0.2 days in ITG1 and ITG2. As for LT_50_, LT_90_ was higher than 20 days in CG1, CG2, ICG1, and ICG2 (Table 2).

**Table 2:** Lethal time needed to *Beauveria bassiana* to kill 50% (LT_50_) and 90% (LT_90_) of mites in different treated and control groups. Superscript letters indicate groups of values without statistically significant differences (ratio test -Wheeler et al. 2006)

*Dermanyssus gallinae* life stage	Lethal time (± SE); days	Without pre-incubation				With pre-incubation			
		TG1	TG2	CG1	CG2	ITG1	ITG2	ICG1	ICG2
Adult	LT_50_	8.8 ± 0.1	9.5 ± 0.1	15.5 ± 0.2	> 20	6.7 ± 0.1	7.4 ± 0.1	16 ± 0.3	> 20
	LT_90_	14.0 ± 0.2	14.9 ± 0.2	> 20	> 20	12.2 ± 0.2^a, b^	12.7 ± 0.2^b, d^	> 20	> 20
Nymph	LT_50_	10.7 ± 0.2	8.6 ± 0.1	> 20	> 20	7.2 ± 0.1	8.0 ± 0.1	19 ± 0.5	> 20
	LT_90_	17.9 ± 0.3	14.4 ± 0.2	> 20	> 20	12.7 ± 0.2^a, c^	13.3 ± 0.2^c, d^	> 20	> 20

## Discussion

Our results show that *B. bassiana* delivered through HEC was efficacious for the control of PRM; the hydrogel delivery system increased the acaricidal activity of *B. bassiana*, mainly when the conidial suspension of 10^7^ conidia/mL *B. bassiana* and HEC were pre-incubated for 4 days at 25 °C. The increase in acaricidal activity of *B. bassiana* delivered through HEC is likely due to the micro-environmental conditions (i.e., pH, high humidity levels, free water, growth humidity, and nutrient availability) favouring the *B. bassiana* growth as previously reported for *Aedes albopictus* (Friuli et al. 2024). To date laboratory research provided solid evidence that EPFs represent a next-generation control tool to manage mosquito, mite and tick populations. In addition, earlier research showed that the delivery system of EPFs increases their viability and infectivity (Cafarchia et al. 2022). However, most of these delivery systems are mainly produced using synthetic and not-biodegradable/renewable polymers for their structure (e.g. polyethylene) and for the preparation of the pesticide release system (e.g., polyacrylamide gels, acrylate resins). In this study, we employed biocompatible biopolymers, an hydrogel derived from natural polymers, particularly polysaccharides, which have gained substantial attention from the scientific community due to their biocompatibility, non-toxicity, biodegradability, bio-stability, and functionalization (Liao and Huang 2020; Huang et al. 2023; Sarwar et al. 2023), and suggest its useful to control PRM infestations. Natural hydrogels based on polysaccharides have been applied in different fields, such as cell/drug delivery, gene delivery, cell culture, and tissue engineering (Varghese et al. 2020; Dattilo et al. 2023). Additionally, HEC has been reported as a useful culture medium of *B. bassiana* (Friuli et al. 2021), as this EPF produces cellulolytic enzymes degrading hydroxyethyl methylcellulose, eventually favoring its growth (Swathi et al. 2018; Friuli et al. 2021).

The higher mortality of *D. gallinae* in CG2 (i.e., HEC hydrogel alone) compared to CG1 (i.e., filter paper alone) suggests that mites may be impaired in their movements, thus reducing their overall fitness and favouring the exposition to *B. bassiana* when the fungus is delivered through HEC. *Beauveria bassiana* has dual pathogenic effect exerted by asexual spores, consisting in the adhesion to the mite cuticle, followed by their germination on it (Liu et al. 2010; Mondal et al. 2016). In addition, the HEC medium may enhance its pathogenic effects, as suggested by the lower lethal time against *D. gallinae* in pre-incubated experimental groups. The treatment with a pre-incubation time can boost the efficacy of *B. bassiana* in HEC, thus overcoming the major limitations in its field use, which is represented by the high amount of entomopathogenic fungal conidia and the long time (i.e., LT_90_ > 14 days) needed to exert the deleterious effect against *D. gallinae* (Immediato et al. 2015; Ebani and Mancianti 2021).

On the other hand, the higher susceptibility to *B. bassiana* of adult *D. gallinae* compared to nymphs is well-known (Immediato et al. 2015; Samish et al. 2001) and it has also been confirmed when HEC-based hydrogel was used as a matrix for *B. bassiana*. The possible explanation for such a sensibility is the thinner structure of nymph cuticle, which may enhance the fungus effect, a factor previously considered by other authors (Wu et al. 2014; Park et al. 2023).

## Conclusion

Overall, our results indicate that HEC-based hydrogel is a suitable delivery substrate for supporting the activity of *B. bassiana* as acaricide against *D. gallinae.* This system is also simultaneously effective against PRM through a combined mechanism of mechanical effect and fungal action. *Beauveria bassiana* can grow and survive in the hydrogel, and this enhances its possibility of reaching the targeted mite pests. At least from a theoretical point of view, this may help in limiting the non-target effects on animal health and the environment and further studies will be aimed to ascertain that. In addition, the pre-incubation of HEC-based hydrogel with *B. bassiana* allows us to use lower conidial concentrations of *B. bassiana* to overcome the obstacles related to mass production to obtain conidia/ propagules resistant or tolerant to the environmental stresses finally resulting a cost-competitive system for precision pest management. The synergistic effects of *B. bassiana* and potential active molecules in the hydrogel suspension may also reduce the lethal time and enhance the specificity against *D. gallinae*, and this will be another target of the future research, since this approach may represent a key step toward addressing concerns associated with the use of chemical acaricides, aiming to reduce environmental and ecological risks associated with mite management strategies in poultry farming.

## Data Availability

We declare all data is being provided within this manuscript.

## References

[CR1] Abbas RZ, Colwell DD, Iqbal Z, Khan A (2013) Acaricidal drug resistance in poultry red mite (*Dermanyssus Gallinae*) and approaches to its management. Worlds Poult Sci J 70:113–124. 10.1017/S0043933914000105

[CR41] *Aspergillus oryzae* (Ahlb.) Cohn (1884). Jber Schles Ges Vaterl Kultur 61:227

[CR39] *Beauveria bassiana *(Bals.-Criv.) Vuill. Bull Soc Bot Fr 59:40 (1912)

[CR2] Cafarchia C, Immediato D, Iatta R, Ramos RA, Lia RP, Porretta D, Figueredo LA, Dantas-Torres F, Otranto D (2015) Native strains of *Beauveria bassiana* for the control of *Rhipicephalus sanguineus* Sensu Lato. Parasit Vectors 8:80. 10.1186/s13071-015-0693-9 25651851 10.1186/s13071-015-0693-9 PMC4324834

[CR3] Cafarchia C, Pellegrino R, Romano V, Friuli M, Demitri C, Pombi M, Benelli G, Otranto D (2022) Delivery and effectiveness of entomopathogenic fungi for mosquito and tick control: current knowledge and research challenges. Acta Trop 234:106627. 10.1016/j.actatropica.2022.106627 35914564 10.1016/j.actatropica.2022.106627

[CR4] Chen Y (2020) Hydrogels based on natural polymers for medicinal applications. Curr Med Chem 27:2608–2609. 10.2174/092986732716200604094035 32515308 10.2174/092986732716200604094035

[CR5] Dattilo M, Patitucci F, Prete S, Parisi OI, Puoci F (2023) Polysaccharide-based hydrogels and their application as drug delivery systems in cancer treatment: a review. J Funct Biomater 14:55. 10.3390/jfb14020055 36826854 10.3390/jfb14020055 PMC9966105

[CR6] De Luna CJ, Arkle S, Harrington D, George DR, Guy JH, Sparagano OA (2008) The poultry red mite *Dermanyssus Gallinae* as a potential carrier of vector-borne diseases. Ann N Y Acad Sci 1149:255–258. 10.1196/annals.1428.085 19120224 10.1196/annals.1428.085

[CR7] de Oliveira DGP, Kasburg CR, Alves LFA (2020) Efficacy of *Beauveria bassiana* against the poultry red mite, *Dermanyssus gallinae* (De Geer, 1778) (Mesostigmata: Dermanyssidae), under laboratory and hen house conditions. Syst Appl Acarology 5:895–905

[CR8] Di Palma A, Giangaspero A, Cafiero MA, Germinara GS (2012) A gallery of the key characters to ease identification of *Dermanyssus gallinae* (Acari: Gamasida: Dermanyssidae) and allow differentiation from *Ornithonyssus Sylviarum* (Acari: Gamasida: Macronyssidae). Parasit Vectors 5:104. 10.1186/1756-3305-5-104 22647594 10.1186/1756-3305-5-104 PMC3419681

[CR9] Ebani VV, Mancianti F (2021) Entomopathogenic fungi and bacteria in a veterinary perspective. Biology 10:479. 10.3390/biology10060479 34071435 10.3390/biology10060479 PMC8229426

[CR11] Friuli M, Nitti P, Aneke CI, Demitri C, Cafarchia C, Otranto D (2021) Freeze-drying of *Beauveria bassiana* suspended in hydroxyethyl cellulose based hydrogel as possible method for storage: evaluation of survival, growth and stability of conidial concentration before and after processing. Results Eng 12:100283. 10.1016/j.rineng.2021.100283

[CR10] Friuli M, Lia RP, Nitti P, Lamanna L, Otranto D, Pombi M, Demitri C, Cafarchia C (2024) Beauveria bassiana associated with a novel biomimetic hydrogel to control *Aedes albopictus* through lure and kill ovitraps. Pest Manag Sci. 10.1002/ps.8476 39415668 10.1002/ps.8476 PMC11716357

[CR12] Hartzke D, Pössl A, Schlupp P, Runkel FE (2022) Evaluation of hydroxyethyl cellulose grades as the main matrix former to produce 3D-printed controlled-release dosage forms. Pharmaceutics 14:2103. 10.3390/pharmaceutics14102103 36297538 10.3390/pharmaceutics14102103 PMC9609046

[CR13] Huang KX, Zhou LY, Chen JQ, Peng N, Chen HX, Gu HZ, Zou T (2023) Applications and perspectives of quaternized cellulose, chitin and chitosan: a review. Int J Biol Macromol 242:124990. 10.1016/j.ijbiomac.2023.124990 37211070 10.1016/j.ijbiomac.2023.124990

[CR14] Immediato D, Camarda A, Iatta R, Puttilli MR, Ramos RA, Di Paola G, Giangaspero A, Otranto D, Cafarchia C (2015) Laboratory evaluation of a native strain of *Beauveria bassiana* for controlling *Dermanyssus gallinae* (De Geer, 1778) (Acari: Dermanyssidae). Vet Parasitol 212:478–482. 10.1016/j.vetpar.2015.07.004 26206607 10.1016/j.vetpar.2015.07.004

[CR15] Immediato D, Figueredo LA, Iatta R, Camarda A, de Luna RLN, Giangaspero A, Brandão-Filho SP, Otranto D, Cafarchia C (2016) Essential oils and Beauveria bassiana against Dermanyssus gallinae (Acari: Dermanyssidae): towards new natural acaricides. Vet Parasitol 229:159–165. 10.1016/j.vetpar.2016.10.018 27809973 10.1016/j.vetpar.2016.10.018

[CR16] Kasburg C, Alves L, Oliveira D, Rohde C (2016) Activity of some Brazilian isolates of entomopathogenic fungi against the poultry red mite *Dermanyssus Gallinae* De Geer (Acari: Dermanyssidae). Braz J Poult Sci 18:457–460. 10.1590/1806-9061-2015-0120

[CR17] Liao J, Huang H (2020) Review on magnetic natural polymer constructed hydrogels as vehicles for drug delivery. Biomacromolecules 21:2574–2594. 10.1021/acs.biomac.0c00566 32543834 10.1021/acs.biomac.0c00566

[CR18] Liu Z, Lei Z, Hua B, Wang H, Liu TX (2010) Germination behavior of *Beauveria bassiana* (Deuteromycotina: Hyphomycetes) on *Bemisia tabaci* (Hemiptera: Aleyrodidae) nymphs. J Entomol Sci 45:322–334. 10.18474/0749-8004-45.4.322

[CR19] Marangi M, Morelli V, Pati S, Camarda A, Cafiero MA, Giangaspero A (2012) Acaricide residues in laying hens naturally infested by red mite *Dermanyssus Gallinae*. PLoS ONE 7:e31795. 10.1371/journal.pone.0031795 22363736 10.1371/journal.pone.0031795 PMC3283649

[CR40] *Metarhizium anisopliae* (Metschn.) Sorokīn (1983) Rastitel’nye Parazity Cheloveka I Zhivotnykh Kak Prichina Zarazhykh Bolezneĭ (Petersburg) 2:268

[CR20] Mondal S, Baksi S, Koris A, Vatai G (2016) Journey of enzymes in entomopathogenic fungi. Pac Sci Rev Nat Sci Eng 18:85–99. 10.1016/j.psra.2016.10.001

[CR21] Moss WW (1968) An illustrated key to the species of the acarine genus *Dermanyssus* (Mesostigmata: Laelapoidea: Dermanyssidae). J Med Entomol 5:67–84. 10.1093/jmedent/5.1.67 5644463 10.1093/jmedent/5.1.67

[CR23] Park SE, Lee MR, Lee SJ, Kim JC, Parker BL, Ryu KS, Lim CI, Kim JS (2022) Strategic positioning of *Beauveria bassiana* sensu lato JEF-410 in management of poultry red mite, *Dermanyssus gallinae*. Biocontrol 67:39–48. Mesostigmata: Dermanyssidae10.1007/s10526-021-10110-w

[CR22] Park SE, Kim JC, Im Y, Kim JS (2023) Pathogenesis and defense mechanism while *Beauveria bassiana* JEF-410 infects poultry red mite, *Dermanyssus gallinae*. PLoS ONE 18:e0280410. 10.1371/journal.pone.0280410 36800366 10.1371/journal.pone.0280410 PMC9937463

[CR24] Ruiu L, Jehle JA, Quesada Moraga E, Tarasco E, Benelli G (2024) Entomopathogens: theory and practice. Crop Prot 184:106813. 10.1016/j.cropro.2024.106813

[CR25] Samish M, Gindin G, Alekseev E, Glazer I (2001) Pathogenicity of entomopathogenic fungi to different developmental stages of *Rhipicephalus sanguineus* (Acari: Ixodidae). J Parasitol 87:1355–1359. 10.1645/0022-3395(2001)087[1355:POEFTD]2.0.CO;2 11780821 10.1645/0022-3395(2001)087[1355:POEFTD]2.0.CO;2

[CR26] Sarwar MS, Ghaffar A, Huang Q, Khalid M, Anwar A, Alayoubi AM, Latif M (2023) Controlled drug release contenders comprising starch/poly(allylamine hydrochloride) biodegradable composite films. Int J Biol Macromol 241:124598. 10.1016/j.ijbiomac.2023.124598 37119890 10.1016/j.ijbiomac.2023.124598

[CR27] Schiavone A, Pugliese N, Otranto D, Samarelli R, Circella E, De Virgilio C, Camarda A (2022) *Dermanyssus gallinae*: the long journey of the poultry red mite to become a vector. Parasit Vectors 15:29. 10.1186/s13071-021-05142-1 35057849 10.1186/s13071-021-05142-1 PMC8772161

[CR28] Schiavone A, Price DRG, Pugliese N, Burgess STG, Siddique I, Circella E, Nisbet AJ, Camarda A (2023) Profiling of Dermanyssus gallinae genes involved in acaricide resistance. Vet Parasitol 319:109957. 10.1016/j.vetpar.2023.109957 10.1016/j.vetpar.2023.109957 37207568

[CR29] Shang J, Tang G, Lu M, Wang C (2022) Host and environmental sensing by entomopathogenic fungi to infect hosts. Curr Clin Microbiol Rep 9:69–74. 10.1007/s40588-022-00185-z

[CR30] Sigognault Flochlay A, Thomas E, Sparagano O (2017) Poultry red mite (Dermanyssus Gallinae) infestation: a broad impact parasitological disease that still remains a significant challenge for the egg-laying industry in Europe. Parasit Vectors 10(357). 10.1186/s13071-017-2292-4 10.1186/s13071-017-2292-4 PMC5537931 28760144

[CR31] Swathi P, Ganga Visalakshy PN, Das SB (2018) In vitro evaluation for compatibility of additives with *Beauveria bassiana* (Balsamo) Vuillemin. Egypt J Biol Pest Control 28:13. 10.1186/s41938-017-0017-9

[CR32] Tomer H, Blum T, Arye I, Faigenboim A, Gottlieb Y, Ment D (2018) Activity of native and commercial strains of *Metarhizium* spp. against the poultry red mite *Dermanyssus gallinae* under different environmental conditions. Vet Parasitol 262:20–25. 10.1016/j.vetpar.2018.09.010 30389007 10.1016/j.vetpar.2018.09.010

[CR33] Truong AT, Yoo MS, Woo SD, Lee H, Park Y, Nguyen TT, Youn SY, Min S, Lim J, Yoon SS, ChoYS (2024) Evaluation of acaricidal activity in entomopathogenic fungi for poultry red mite (*Dermanyssus Gallinae*) control. Vet Parasitol 331:110292. 10.1016/j.vetpar.2024.110292 39208531 10.1016/j.vetpar.2024.110292

[CR34] Varghese SA, Rangappa SM, Siengchin S (2020) Natural polymers and the hydrogels prepared from them. In: Chen Y (ed) Hydrogels based on natural polymers. Elsevier, Amsterdam, pp 17–47. 10.1016/B978-0-12-816421-1.00002-1

[CR35] Wang C, Huang Y, Zhao J, Ma Y, Xu X, Wan Q, Li H, Yu H, Pan B (2019) First record of *aspergillus oryzae* as an entomopathogenic fungus against the poultry red mite *Dermanyssus Gallinae*. Vet Parasitol 271:57–63. 10.1016/j.vetpar.2019.06.011 31303205 10.1016/j.vetpar.2019.06.011

[CR36] Wheeler MW, Park RM, Bailer AJ (2006) Comparing median lethal concentration values using confidence interval overlap or ratio tests. Environ Toxicol Chem 25:1441–1444. 10.1897/05-320r.1 16704080 10.1897/05-320r.1

[CR37] Wu S, Gao Y, Zhang Y, Wang E, Xu X, Lei Z (2014) An entomopathogenic strain of *Beauveria bassiana* against *Frankliniella occidentalis* with no detrimental effect on the predatory mite *Neoseiulus barkeri*: evidence from laboratory bioassay and scanning electron microscopic observation. PLoS ONE 9:e84732. 10.1371/journal.pone.0084732 24454744 10.1371/journal.pone.0084732 PMC3891770

